# Modified sharp regression discontinuity model to settings with fuzzy variables

**DOI:** 10.1186/s13104-023-06561-2

**Published:** 2023-10-18

**Authors:** Portia K. Mafukidze, Samuel M. Mwalili, Thomas Mageto

**Affiliations:** 1 Department of Mathematics, The Pan African University, Institute for Basic Sciences, Technology and Innovation, Nairobi, Kenya; 2 Department of Statistics and Actuarial Sciences, Jomo Kenyatta University of Agriculture and Technology, Nairobi, Kenya

**Keywords:** Sharp regression discontinuity model, People Living with HIV and AIDS, Fuzzy variables, AUDIT score, CD4 Counts, Viral loads

## Abstract

**Objective:**

The goal of this study is to develop a Modified Sharp Regression Discontinuity model to predict alcohol consumption in People Living with Human Immunodeficiency Virus (HIV) and Acquired Immunodeficiency Syndrome (AIDS). Previous studies focused on either fuzzy dependent or fuzzy independent variables separately. However, there is a gap in research that examines the interaction between both types of fuzzy variables thus the model considers both dependent and independent fuzzy variables.

**Methods:**

A statistical model was developed to predict the relationship between alcohol consumption and HIV progression. The model equations are solved numerically using parametric estimation.

**Results:**

In simulation studies, as the sample size expanded, the estimates derived from the modified sharp regression discontinuity model exhibited probabilistic convergence towards the true value, thereby validating the estimator of the Average Causal Effect’s consistency. Counseling has an average causal effect in the sharp Regression Discontinuity Design (RDD) for compliers that is roughly equal to 0.199. This was the variation in Alcohol Use Detective Identification Test (AUDIT) threshold scores or the change in intercept scores when counseling was effective. Following six months of participation in the counseling program, AUDIT scores decreased, leading to an increase in Cluster of Differentiation 4 (CD4) counts and a decrease in viral loads.

**Conclusion:**

The Modified Sharp RDD offers a robust approach to handle fuzzy variables in causal inference. Our study contributes to the advancement of RDD methodology and its applicability in real-world settings with uncertain data.

## Introduction

In this research study, a total of 234 individuals were initially recruited. However, during the follow-up at 3 and 6 months, 44 participants were lost, resulting in a final sample size of 190 participants. The data for this study was collected from 16 HIV care clinics in Zimbabwe, which were selected using cluster sampling. These public health facilities were chosen on a national scale using a combination of stratified randomization and random allocation methods. Participants who scored above 15 or equal to 15 on the AUDIT (Alcohol Use Disorders Identification Test) were identified as individuals with alcohol dependence and received counseling sessions.

The prediction of alcohol consumption in People Living with Human Immunodeficiency Virus (HIV) and Acquired Immunodeficiency Syndrome (AIDS) is a significant concern for public health. Sharp Regression Discontinuity Design (RDD) has proven useful for analyzing causal effects in such settings. However, traditional RDD models assume crisp data, while real-world data often involve uncertainty or fuzziness. To address this limitation, a Modified Sharp Regression Discontinuity model that accounts for both dependent and independent fuzzy variables is proposed.

The regression discontinuity design (RDD) is said to be sharp if the likelihood of being treated grows from zero to one, otherwise, it is said to be fuzzy. RDD is crisp when all individuals receive the planned therapy [1].

According to [2], in a strong regression discontinuity, the likelihood of receiving treatment jumps deterministically from 0 to 1 at the cut-off. Everyone on one side of the cutoff gets treatment, but no one on the other gets it. In the abrupt regression discontinuity, the treatment impact is assessed by comparing outcomes for those immediately above and below the cutoff.

In the context of HIV and AIDS patient data, fuzzy variables play a crucial role, particularly when dealing with viral load and immune system strength. The fuzzy nature of these variables makes the traditional RDD less effective in capturing the complex relationships within the data [3]. To address this limitation, our study proposed a Modified Sharp RDD that can handle both dependent and independent fuzzy variables. Using this model with an AUDIT score-based cutoff, we investigated the effects of alcohol usage on viral loads via CD4 counts in HIV and AIDS patients (PLWHA). An AUDIT score of 15 is regarded as the cutoff.

The immune system’s strength or weakness determines the dependent variable viral load decrease or increase in the body, which differs from person to person. T-cell depletion is less common in patients or anyone with a healthy immune system, but it is more common in persons who have a weak immune system. This implies that the variable viral load is uncertain or fuzzy. As a result, the amount or quantity of immune cells and the HIV viral load at various stages of the disease can be regarded as unclear [3].

## Main text

### Methods

In the study, a statistical model for predicting alcohol consumption and HIV progression is formulated. We considered a case in which AUDIT scores and CD4 counts are considered fuzzy. The Modified Sharp Regression Discontinuity model incorporates imprecise observations. Studies have considered cases in which the observations are clear. In actual fact this is not always the case since the variables involved will be fuzzy at times.

To calculate the result variable’s discontinuity at the cut-off point.

1 $$ \mathop {\lim }\limits_{{{\mathbf{x}} \downarrow {\mathbf{x^{\prime}}}}} E(y_{{ij}} |x_{{ij}}  = x) - \mathop {\lim }\limits_{{{\mathbf{x}} \uparrow {\mathbf{x^{\prime}}}}} E(y_{{ij}} |x_{{ij}}  = x) $$

This is equal to the Average Causal Effect (ACE), i.e.,

2 $$ ACE = \delta _{{SRD}}  = E(y_{{ij}} (1) - y_{{ij}} (0)|x_{{ij}}  = x^{\prime}) $$

The treatment impact for a specific subpopulation $x_{ij}=x'$ is denoted by $\delta _{SRD}$

We can define conditional means from the right and left respectively as follows:

3 $$ \mu _{a} (x^{\prime}) = \mathop {\lim }\limits_{{{\mathbf{x}} \downarrow {\mathbf{x^{\prime}}}}} E(y_{{ij}} |x_{{ij}}  = x) $$

4 $$ \mu _{b} (x^{\prime}) = \mathop {\lim }\limits_{{{\mathbf{x}} \uparrow {\mathbf{x^{\prime}}}}} E(y_{{ij}} |x_{{ij}}  = x)  $$

These two can be estimated separately and then take the difference or just find the difference between them at once. Considering the earlier we can solve

5 $$\begin{aligned} \min _{{{\alpha _b}{\beta _b}}} \sum _{ij|x'-h<x_{ij}\le x'}^{}(y_{ij}-\alpha _b-\beta _b(x_{ij}-x'))^{2} \end{aligned}$$

as well as

6 $$ \min _{{\alpha _{a} \beta _{a} }} \sum\limits_{{ij|x^{\prime}<x_{{ij}}< x^{\prime} + h}}^{{}} {(y_{{ij}}  - \alpha _{a}  - \beta _{a} (x_{{ij}}  - x^{\prime}))^{2} }  $$

which results in

7 $$ \hat{\mu }_{b} (x^{\prime}) = \widehat{{\alpha _{b} }} + \widehat{{\beta _{b} }}(x^{\prime} - x^{\prime}) = \widehat{{\alpha _{b} }} $$

and

8 $$ \hat{\mu }_{a} (x^{\prime}) = \widehat{{\alpha _{a} }} + \widehat{{\beta _{a} }}(x^{\prime} - x^{\prime}) = \widehat{{\alpha _{a} }} $$

The difference between estimates in Eq.(8) and Eq. (7) is the size of the discontinuity that is

9 $$ \hat{\alpha _{a}}-\hat{\alpha _{b}} $$

If $$x_{ij}<x'$$

10 $$E(y_{ij}|x_{ij}=x)=\alpha _{a}+\beta _{a}(x_{ij}-x') +e_{ij} $$

and if $$x_{ij}\ge x'$$

11 $$E(y_{ij}|x_{ij}=x)=\alpha _{b}+\beta _{b}(x_{ij}-x')+e_{ij} $$

Or alternatively we can run

12 $$ \min _{{\alpha \beta \gamma \delta }} \sum _{ij|x'-h< x_{ij}<x'+h}^{}(y_{ij}-\alpha -\beta (x_{ij}-x')-\gamma d_{ij}(x_{ij}-x')-\delta d_{ij})^{2} $$

and use the Ordinary Least Squares to estimate the parameters. We had a classical regression of the form:

13 $$ y_{ij}=\alpha +\beta (x_{ij}-x')+\gamma {d_{ij}}(x_{ij}-x')+\delta {d_{ij}}+{e_{ij}}^{*} $$

Since the dependent and independent variables are fuzzy, we have a model of the form

14 $${y_{ij}}^{*}=\alpha +\beta (1-{d_{ij}})({x_{ij}}^{*}-{x'}^{*})+\gamma {d_{ij}}({x_{ij}}^{*}-{x'}^{*})+\delta {d_{ij}}+{e_{ij}}^{**} $$

where $${y_{ij}}^{*}$$ and $${x_{ij}}^{*}$$ are fuzzy observations with membership functions defined as $$\mu _{\overline{y}_{ij}}(y)$$ and $$\mu _{\overline{x}_{ij}}(x)$$ respectively. $$e_{ij}^{**}$$ was the fuzzy error associated with the model. This error term can be estimated using the idea by [4].

NB: Please note that for sharp regression discontinuity we do not have uncompliers to the program as in fuzzy RDD.

Now letting ${x_{ij}}^{*}-{x'}^{*} ={x_{ij}}^{c}$ and $\psi = {(\alpha ,\beta ,\gamma ,\delta )}^{T}$ and considering the matrix

$$X{\mkern 1mu}  = {\mkern 1mu} \left( {\begin{array}{*{20}{c}}
  1&{\left( {1 - \mathop {{d_{1j}}}\limits^ \wedge  } \right)}&{x{{_{1j}^{}}^c}}&{\mathop {{d_{1j}}}\limits^ \wedge  x{{_{1j}^{}}^c}}&{\mathop {{d_{1j}}}\limits^ \wedge  } \\ 
   \vdots & \vdots & \vdots & \vdots & \vdots  \\ 
  1&{\left( {1 - \mathop {{d_{nj}}}\limits^ \wedge  } \right)}&{x{{_{nj}^{}}^c}}&{\mathop {{d_{nj}}}\limits^ \wedge  x{{_{nj}^{}}^c}}&{\mathop {{d_{nj}}}\limits^ \wedge  } 
\end{array}} \right)$$

we have a model in matrix form as

15 $$ {y_{ij}}^{*}=X\psi +{e_{ij}}^{**} $$

When $d_{ij}=0$ then Eq. (14) becomes

16 $$ {y_{ij}}^{*}=\alpha +\beta ({x_{ij}}^{*}-{x'}^{*})+{e_{ij}}^{**} $$

Moreover when $d_{ij}=1$, Eq. (14) becomes

17 $$ {y_{ij}}^{*}=\alpha +\gamma ({x_{ij}}^{*}-{x'}^{*})+\delta +{e_{ij}}^{**}$$

Now solving Eq. (16) and Eq. (17) when ${x_{ij}}^{*}={x'}^{*}$ we have $E({y_{ij}}^{*}|d_{ij}=0,{x_{ij}}^{*}={x'}^{*})=\alpha$ and $E({y_{ij}}^{*}|d_{ij}=1,{x_{ij}}^{*}={x'}^{*})=\alpha +\delta$.

As a result to calculate the treatment effect, $E({y_{ij}}^{*}|d_{ij}=1,{x_{ij}}^{*}={x'}^{*})-E({y_{ij}}^{*}|d_{ij}=0,{x_{ij}}^{*}={x'}^{*})=\delta$.

## Results

### Implementation of the modified sharp regression discontinuity model

**Fig. 1:**
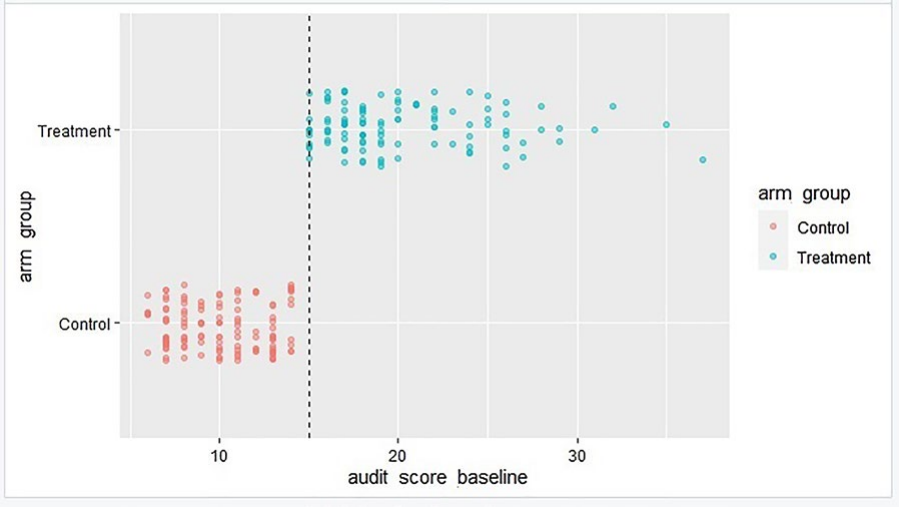
Compliance around the a cutoff

With perfect compliance (100%), we measured the size of the leap at the cutoff, indicating a sharp regression discontinuity. All participants above the cutoff received treatment, while those below it did not. The graphical representation is shown in Fig. 1.

#### Check for discontinuity in the running variable around cutoff point in sharp RDD

To see if the running variable was manipulated, imagine there was a large number of participants clustered around 15 due to how the AUDIT was administered; that is, respondents wanted to get into the program, so they purposefully answered some questions incorrectly. In this case, we did so by making a histogram of the running variable (AUDIT scores) and looking for any significant jumps around the threshold. There was a very slight visible difference in the height of the bars before and after the 15-score cutoff, so there doesn’t appear to be a jump around the threshold in this case. Using a McCrary density test to determine whether that jump was statistically significant, the overlap’s p value is equal to 0.47, indicating that there is no significant difference near the threshold [5].

#### Check for discontinuity in the outcome variable across running variable in sharp RDD

**Fig. 2:**
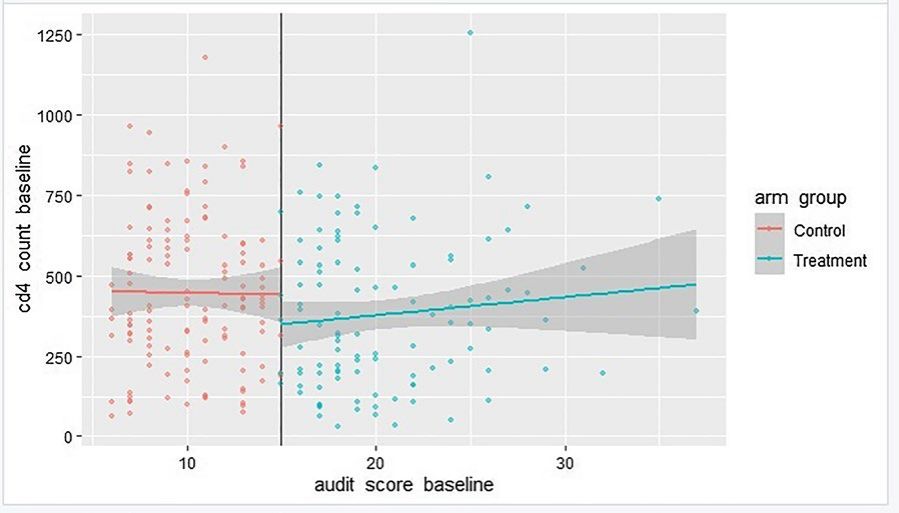
Checking for discontinuity in the sharp outcome variable

We can finally see if there was a discontinuity in the final AUDIT scores based on participation in the counseling program because this is a sharp regression discontinuity design and there was no bunching of AUDIT scores around the 15-point threshold. Figure 2 clearly shows a discontinuity. It appeared that taking part in the counseling program improved the CD4 counts and, consequently, the final AUDIT scores.

**Fig. 3:**
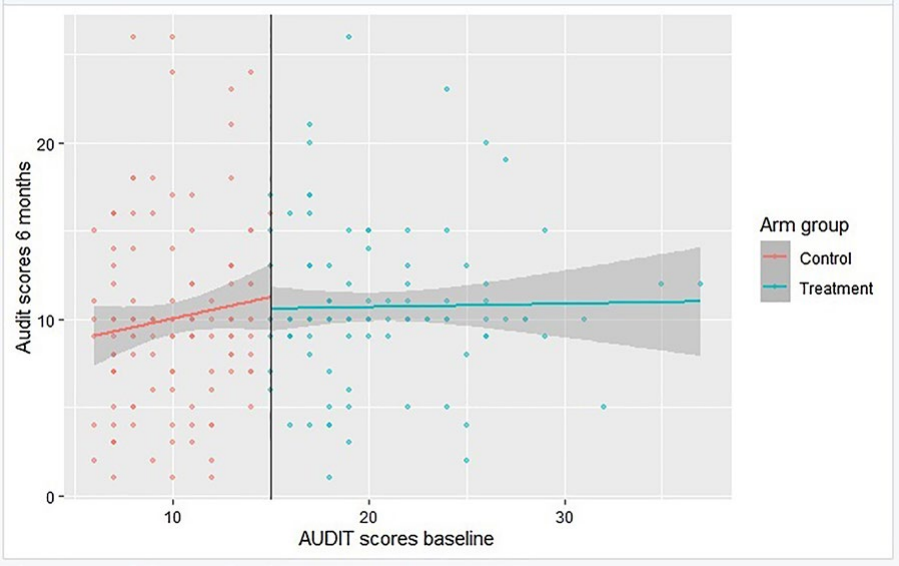
Checking for relationship between AUDIT scores baseline and AUDIT scores 6 months

For the case of AUDIT scores, the improvement can be explained in form of the graph in Figure  3.

### Estimation of the size of the effect in sharp RDD

The coefficient, which takes into account the counseling program, is the one we are most concerned about. This causal effect of counseling for compliers in sharp RDD is approximately equal to 0.199, which is lower than that in fuzzy RDD [6]. In other words a small effect in the positive direction [7]. This was the change in intercept when counseling was accurate or the variance in AUDIT threshold scores. Participating in the counseling program raised AUDIT scores after six months, which raised CD4 counts and lowers viral loads.

###  Simulation of estimates based on modified sharp regression discontinuity model

In this section, we performed simulations to assess the asymptotic properties of the proposed methodology. For this model, using a cutoff $x'=15$, the parameters were set at $\nu =10$, $\beta =0.5$ and $\gamma =0.3$ where $\nu =\alpha +\delta$ and $\eta =(\nu , \beta ,\gamma )^{T}$. Based on 1000 replications, table 1 shows the simulated estimates when n=30, n=50, n=100 and n=200.

**Table 1:** Modified Sharp RDD simulated estimates

n=30	$\eta _{1}$	$\eta _{2}$	$\eta _{3}$	$\eta _{1}$	$\eta _{5}$	$\eta _{6}$	$\eta _{7}$	$\eta _{8}$	$\eta _{9}$	$\eta _{10}$
$\nu$	10.3571	10.13699	10.3151	10.07119	10.14692	9.941882	10.01227	10.03112	10.0799	10.10933
$\beta$	0.423301	0.304745	0.163721	0.962319	0.780591	0.799358	0.524637	0.625527	0.187328	0.162328
$\gamma$	0.70757	0.340816	0.675583	0.212815	0.313959	0.065886	0.092844	0.075818	0.408715	0.561309
n=50										
$\nu$	9.911002	9.872703	10.0042	10.09243	9.991801	9.778196	10.15413	10.08228	9.798289	10.14695
$\beta$	0.5321	0.40882	0.512039	0.556041	0.611638	0.326308	0.659823	0.747967	0.330914	0.514188
$\gamma$	0.3429	0.495762	0.239283	0.426391	0.241694	0.567491	0.075116	0.19708	0.243548	0.350965
n=100										
$\nu$	10.12069	10.12225	9.866842	10.05424	10.14138	9.993583	10.0032	10.07817	10.07679	10.02832
$\beta$	0.587732	0.477938	0.521734	0.479083	0.423869	0.473041	0.645547	0.562808	0.252517	0.276327
$\gamma$	0.105155	0.333136	0.435564	0.30781	0.438764	0.283902	0.315257	0.189087	0.589927	0.409722
n=200										
$\nu$	9.944599	10.08645	9.995438	9.977704	10.03218	10.0959	10.03705	9.924101	9.996316	10.10225
$\beta$	0.434522	0.47874	0.373126	0.639747	0.41859	0.582031	0.470742	0.575179	0.552785	0.536087
$\gamma$	0.372102	0.227076	0.40868	0.063141	0.379626	0.285347	0.353391	0.19462	0.237633	0.17415

**Fig. 4:**
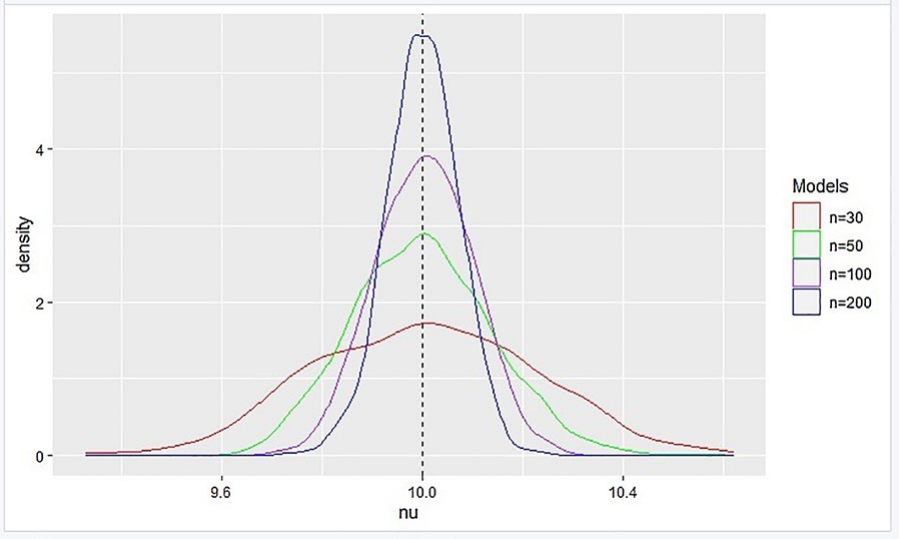
Distribution of sharp estimate $\nu$ as the sample size increases

The subsequent curves in Modified Sharp RDD also exhibited a bell-shaped distribution as in Figure 4.

**Fig. 5:**
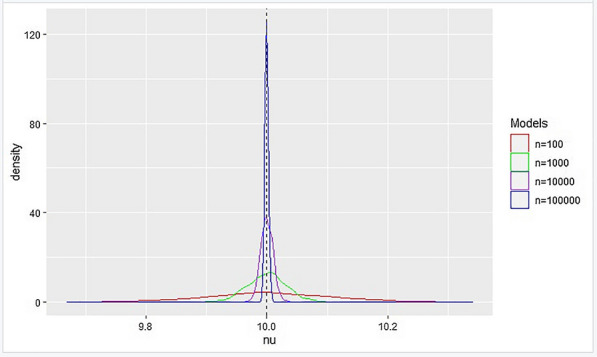
Distribution of sharp estimate $\nu$

As the sample size grew, the estimates derived using the modified sharp regression discontinuity model converged in probability to the true value, proving the consistency of the estimator. Asymptotically consistent estimators in Modified Sharp RDD have produced the curve that is displayed in Fig. 5.

Based on the results from simulation, we can conclude that the estimates from both the Modified Fuzzy and Sharp regression discontinuity models are asymptotically consistent and follow a normal distribution [6].

### Discussion

Our study presents a Modified Sharp Regression Discontinuity model that successfully addresses the challenges posed by fuzzy variables in HIV and AIDS patient data. By incorporating both fuzzy independent and dependent variables, our model offers a more accurate prediction of alcohol consumption and its impact on HIV progression.

In comparing our results to similar studies conducted by other researchers, we find that our Modified Sharp RDD provides more robust estimates for the causal effect of counseling on alcohol consumption among patients living with HIV and AIDS [6]. The estimator demonstrated consistency and asymptotic convergence towards the true value, validating its reliability.

The findings of our simulation studies support the claim that the Modified Fuzzy and Sharp RDD models are asymptotically consistent and follow a normal distribution as discussed by [6]. These results hold promise for improved causal inference in settings with fuzzy variables.

However, we acknowledge some limitations in our study. The external validity of our findings may be restricted due to the inclusion of data from a specific region (Zimbabwe). Therefore, caution should be exercised when generalizing our results to other populations.

In conclusion, our study contributes to the growing body of research on RDD models and provides a valuable framework for addressing fuzzy variables in various applications, particularly in the field of healthcare research. Further research can explore the potential of our Modified Sharp RDD in broader contexts and different datasets.

## Limitations

The inclusion of data from Zimbabwe in this study restricted the external validity of the results. The findings may be reliably extrapolated to the population of PLWHA in Zimbabwe, and as a result, the internal validity is high. However, it is debatable if the Zimbabwean patients (participants) reflect the broader population of people living with HIV and AIDS in Africa and beyond.

## Data Availability

Not Applicable.

## Abbreviations

ACE: Average causal effect
CD4: Cluster of differentiation 4
WHO: World Health Organisation
HIV: Human immunodeficiency virus
RDD: Regression discontinuity design
AIDS: Acquired immunodeficiency syndrome
PLWHA: People living with HIV and AIDS
AUDIT: Alcohol use disorders identification test
